# Femtosecond Laser Ablation of Copper-Hydroxyphosphate-Modified CFRP

**DOI:** 10.3390/ma18214879

**Published:** 2025-10-24

**Authors:** Denys Baklan, Oleksiy Myronyuk, Anna Bilousova, Paulius Šlevas, Justinas Minkevičius, Orestas Ulčinas, Sergej Orlov, Egidijus Vanagas

**Affiliations:** 1 Department of Chemical Technology of Composite Materials, Chemical Technology Faculty, Igor Sikorsky Kyiv Polytechnic Institute, Beresteiskyi Ave. 37, 03056 Kyiv, Ukraine; o.myronyuk@kpi.ua (O.M.); a.bilousova@kpi.ua (A.B.); 2 Coherent Optics Laboratory, Department of Optoelectronics, Center for Physical Sciences and Technology, Sauletekio Ave. 3, LT-10257 Vilnius, Lithuania; paulius.slevas@ftmc.lt (P.Š.); justinas.minkevicius@ftmc.lt (J.M.); orestas.ulcinas@ftmc.lt (O.U.); sergejus.orlovas@ftmc.lt (S.O.); egidijus.vanagas@ftmc.lt (E.V.)

**Keywords:** CFRP, epoxy resin, carbon fiber, laser processing, cutting, ablation, copper hydroxyphosphate

## Abstract

Carbon-fiber-reinforced plastic (CFRP) machining by ultrashort-pulse lasers promises high precision but is limited due to the heterogeneous epoxy–carbon fiber structure, which creates heat-affected zones and variable kerf quality. This work investigates synthesized copper hydroxyphosphate as a laser-absorbing additive to improve femtosecond (1030 nm) laser ablation of CFRP. Copper hydroxyphosphate particles were synthesized hydrothermally and incorporated into an epoxy matrix to produce single-ply CFRP laminates. Square patterns (0.5 × 0.5 mm) were ablated with a pulse energy of 0.5–16 μJ. Then, ablated volumes were profiled and materials characterized by SEM and EDS. In neat epoxy the copper additive reduced optimum ablation efficiency and decreased penetration depth, while producing smoother, less porous surfaces. In contrast, CFRP with copper hydroxyphosphate showed increased efficiency and higher penetration depth. SEM and EDS analyses indicate more uniform matrix removal and retention of resin residues on fibers. These results suggest that copper hydroxyphosphate acts as a local energy absorber that trades volumetric removal for improved surface quality in epoxy and enhances uniformity and process stability in CFRP femtosecond laser machining.

## 1. Introduction

Carbon-fiber-reinforced plastics (CFRPs) are polymer matrix composites that combine high-strength carbon fibers with a thermosetting epoxy resin, yielding materials with a high strength-to-weight ratio and high elastic modulus due to the low density of the fibers and the rigid polymer network [1,2,3]. In addition to their mechanical advantages, CFRPs demonstrate excellent corrosion and chemical resistance, thermal stability, and the capability to form complex geometries. This explains their widespread adoption in aerospace, marine, automotive, construction, and energy applications, for example, in wind turbine blades [4,5,6]. Compared to metallic structural materials, CFRP often provides higher stiffness and strength at a lower mass with superior fatigue and corrosion performance, motivating their increasing use in weight-sensitive engineering structures [7].

Conventional CFRP processing methods include mechanical cutting, milling, drilling, waterjet, and abrasive-jet machining, and are mature and scalable but also have limitations. Mechanical methods cause tool wear, induce microcracks, fiber pull-out, and interlaminar delamination, and generate damage in the polymer matrix [8]. Waterjet and abrasive-jet techniques reduce thermal loading but often produce tapered profiles, require high energy input, and impose costs for abrasive handling and disposal [9]. Laser processing, as a non-contact technique, offers high precision, speed, and repeatability without tool wear and with reduced dust emissions. These advantages make it an attractive alternative for CFRP machining [10,11]. Ultrashort-pulse (femtosecond) lasers can minimize heat diffusion and heat-affected zone (HAZ) size relative to nanosecond and picosecond lasers. This can enable thermal ablation regimes and higher-quality cuts in polymer-based composites [12,13,14].

The interaction between the laser and the CFRP is governed by the optical and thermal contrasts between the fiber and the matrix, as well as by the laser parameters (wavelength, fluence, pulse duration, and repetition rate). Carbon fibers strongly absorb infrared (IR) radiation, whereas typical epoxy matrices exhibit much lower absorption in the IR range. Also, UV laser wavelengths can directly break polymer bonds and promote photochemical mechanisms of ablation [10,15,16]. Pulse duration critically determines the balance between nonthermal (photo-induced) and thermal mechanisms. Nanosecond pulses favor heat accumulation, melting, and a wide HAZ, while picosecond and femtosecond pulses confine energy deposition temporally and reduce collateral heating, improving cut quality when parameters are optimized [17].

Despite these advantages, laser cutting of CFRP still faces practical challenges. Selective epoxy matrix degradation, formation of HAZs, rough cut surfaces, and kerf tapering are frequently reported, all of which can expose fibers, reduce interfacial adhesion, and degrade mechanical performance of the processed part [18,19,20]. Polymer degradation under intense irradiation proceeds via bond scission and pyrolysis pathways that generate small volatile species and carbonaceous residues. These processes are exacerbated by heat conduction along fibers into the surrounding matrix, causing additional local thermal damage and delamination [21,22].

To mitigate matrix damage and improve cut quality, several strategies have been explored. Optimization of laser parameters (reduced pulse energy, increased scanning speed, multi-pass strategies), processing atmosphere control (oxygen or inert gas assist), and implementation of waterjet-guided laser techniques have demonstrated reductions in HAZ size and improved kerf characteristics [14,23,24]. Complementary to these operational measures, polymer matrix modification by incorporation of light-absorbing additives offers a route to redistribute deposited energy, limit localized overheating, and steer the ablation mechanism towards more controlled removal. Such absorbers include inorganic oxides and salts, metal nanoparticles, carbonaceous fillers (carbon black, carbon nanotubes, graphene), and phosphate-based compounds. Hierarchical nanosheet architectures have been shown to markedly increase adsorption capacity and interfacial interaction in related systems, enhancing uptake and surface activity of incorporated species [25]. Experimental studies report that these fillers can reduce char formation, smooth the kerf, and improve fiber–matrix adhesion after laser processing [26,27]. Also, laser-absorbing compounds are often used as additives for laser marking. For example, for the 1064 nm laser wavelength, antimony-doped tin oxide [28] or diantimony trioxide [29] are used. But most effective for the 1030 nm or 1064 nm lasers are copper salts [30]. For example, a copper phosphate (Cu3P2O8) exhibits broadband Vis-NIR absorption (500–2200 nm) and a high photon-to-heat conversion efficiency but low thermal conductivity (0.64 W/m·K) [31]. By contrast, copper hydroxyphosphate (Cu_2_(OH)PO_4_) particles show broad UV-Vis-NIR absorption with a main peak around 530–540 nm and continued absorption into the near-IR range up to 900 nm [32]. Direct photothermal conversion efficiency for copper hydroxyphosphate in epoxy has not yet been reported. Carbon-based absorbers (carbon black, carbon nanotubes) absorb across more than 80% of the NIR spectrum [33] and convert a large fraction of light to heat [34]. Metal oxides, for example, titanium dioxide, have moderate NIR absorption and intermediate thermal conductivities (~10–50 W/m·K depending on the temperature) [35,36].

Copper hydroxyphosphate (Cu_2_(OH)PO_4_) was demonstrated as a near-infrared-absorbing additive for plastic laser marking and it is a promising laser absorber for epoxy-based composites. It has a suitable NIR absorption range but has moderate thermal conductivity [37]. Structurally, Cu_2_(OH)PO_4_ consists of alternating Cu octahedron and trigonal bipyramid coordination units and PO_4_ tetrahedra with bridging hydroxyls, which yields notable optical activity and thermal stability [38,39]. Copper hydroxyphosphate and related copper phosphates exhibit absorption bands extending into the visible and near-infrared region and can be synthesized by straightforward wet-chemical or hydrothermal routes, which facilitates their incorporation as micro-/nano-fillers in an epoxy matrix [40,41,42]. In addition, wet and hydrothermal methods yield a product suitable for further processing and incorporation into a matrix, as well as providing control over particle morphology and dispersion, which is critical for controlled energy absorption and transport during laser exposure [43]. The combination of favorable spectral response, chemical robustness, and intrinsic thermal transport of copper-based phases suggests that Cu_2_(OH)PO_4_ may serve as an efficient local energy absorber for laser irradiation, moderating matrix heating and improving material removal homogeneity.

In summary, while advances in ultrashort-pulse laser processing and parameter control have improved CFRP machining, epoxy matrix thermal degradation and the resulting defects continue to limit cut quality and structural performance. The targeted introduction of laser-absorbing fillers, particularly copper hydroxyphosphate, represents a promising strategy to redistribute laser energy, reduce localized damage, and enhance process reproducibility. Motivated by these considerations, the present study focuses on the synthesis of copper hydroxyphosphate particles and their incorporation into epoxy and CFRP systems, with the aim of assessing their influence on femtosecond laser ablation efficiency, kerf morphology, and the uniformity of matrix removal.

## 2. Materials and Methods

### 2.1. CFRP Materials and Copper Hydroxyphosphate Synthesis

To obtain CFRP, the epoxy resin CHS-EPOXY 619 (viscosity 0.4–0.9 Pa·s, 25 °C, EEW 155–170) and cycloaliphatic hardener TELALIT 0600 (HEW 62 g/mol, amine number 450–500 mg KOH/g) were used. Both were purchased from a local supplier (Spolchemie, Usti nad Labem, Czech Republic). Carbon fabric 3K, 200 g/m^2^, twill, was used for reinforcing fibers.

**Figure 1:**
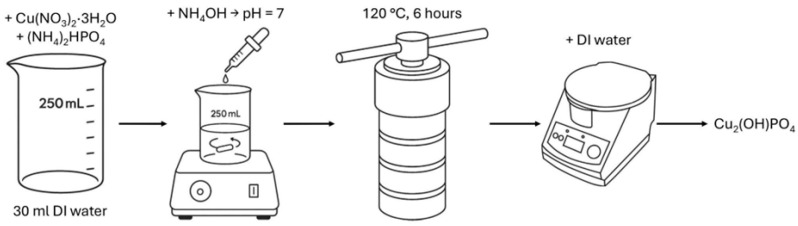
Scheme of copper hydroxyphosphate synthesis procedure.

Copper hydroxyphosphate (Cu_2_(OH)PO_4_) was synthesized using a modified hydrothermal method (Figure 1), based on papers [27,42,44,45]. Initially, 0.02 mol of copper (II) nitrate trihydrate (Cu(NO_3_)_2_·3H_2_O) and 0.01 mol of diammonium phosphate ((NH_4_)_2_HPO_4_) were dissolved in 30 mL of deionized water. Copper (II) nitrate trihydrate was chosen as the precursor owing to the high photocatalytic activity of Cu_2_(OH)PO_4_ synthesized from this compound [45]. The solution pH was adjusted to 7 by the dropwise addition of aqueous ammonia, followed by continuous stirring with a magnetic stirrer for 10 min. All named substances were purchased from the local supplier HLR Ukraine (Chemlaborreactiv LLC, Brovary, Ukraine). The resulting mixture was then transferred into a polytetrafluoroethylene-lined stainless-steel autoclave (BAOSHISHAN, Zhengzhou, China) and subjected to hydrothermal treatment at 120 °C for 6 h. The obtained precipitate was collected, thoroughly washed with deionized water, separated by centrifugation (CF-10, Daihan Scientific, Daejeon, Republic of Korea), and subsequently dried at 60 °C for 2 h to yield the copper hydroxyphosphate product.

### 2.2. CFRP Preparation

Carbon-fiber-reinforced plastic (CFRP) laminates were manufactured using an epoxy resin matrix modified with a copper hydroxyphosphate additive. A pre-weighed amount of copper hydroxyphosphate powder (1 wt. %) was dispersed in the epoxy oligomer by mechanical stirring at 2000 rpm for 10 min. The mixture was then vacuumed to remove entrapped air bubbles. After dispersion, a curing agent was added in the stoichiometric ratio recommended by the supplier, and the mixture was stirred for another 5 min at 200 rpm.

**Figure A1:**
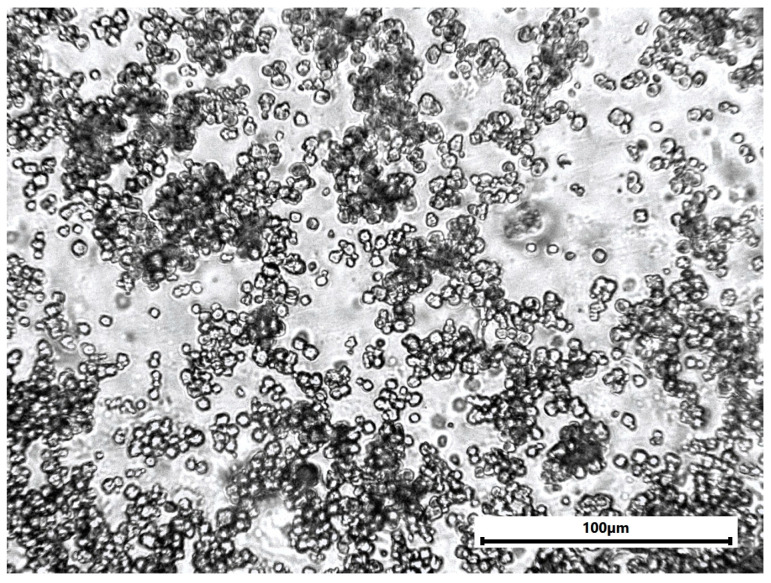
Optical microscopy of epoxy sample with copper hydroxyphosphate particles.

The carbon fiber fabric was impregnated with the epoxy system by hand lay-up and compressed under pressure to expel air bubbles. The laminates were made from 1 layer with a fiber volume fraction of approximately 60%. The curing process was carried out at room temperature for 72 h, followed by post-curing at 50 °C for 2 h to ensure complete epoxy resin system cross-linking. Copper hydroxyphosphate powder was evenly distributed throughout the epoxy resin (Figure A1). The prepared CFRP laminates were cut into samples of sizes suitable for femtosecond laser ablation experiments (50 × 30 mm).

### 2.3. Optical Setup

**Figure 2:**
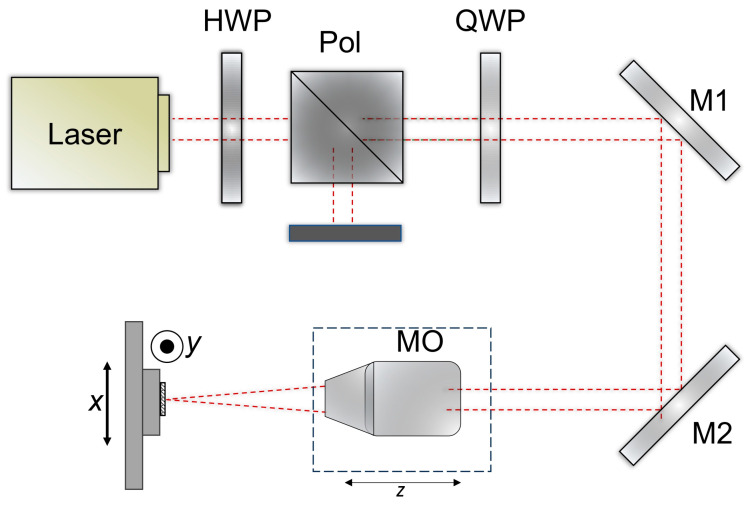
Optical setup for the CFRP laser ablation.

The optical setup is shown in Figure 2. A laser beam with a wavelength of 1030 nm (Pharos laser, Light Conversion, Vilnius, Lithuania) and a diameter of approximately 4.1 mm (1/e^2^) was passed through a quarter-wave plate (QWP) and directed into a 10× Plan Apo NIR microscope objective (MO) (10× Mitutoyo Plan Apo NIR, Kawasaki, Japan) mounted on a motorized z-stage. The power was controlled by an external attenuator comprising a half-wave plate (HWP) and a polarizer (Pol). The beam was focused on a spot size of ~3.9 μm on the CFRP sample surface, which was fixed on XY linear stages (Aerotech ant130-xy, Pittsburgh, PA, USA). The laser system could operate at powers up to 6 W and repetition rates up to 200 kHz. The output power was further adjusted using an external attenuator consisting of a half-wave plate (HWP) and a polarizer. Optimal parameters were evaluated in previous work [46].

During the experiments, the CFRP samples were processed by a stage bidirectional hatching motion, with a hatch spacing of 1.4 μm and a pulse-to-pulse overlap of ~82% along each line for the best pulse duration and fluence. Square patterns of 0.5 × 0.5 mm were fabricated without repeating the hatch. The single-pulse energy applied within the squares ranged from 0.5 μJ to 16 μJ. Pulse duration was determined by the shortest pulse duration available for a femtosecond laser—190 fs. After laser processing, the samples were cleaned in an ultrasonic bath using distilled water. The ablated square volumes were subsequently measured with an optical profiler (Sensofar S neox, Barcelona, Spain), and the energy-specific volume was then calculated.

### 2.4. Characterization

Chemical analysis was performed via FTIR in ATR mode on an IRSpirit spectrometer (Shimadzu, Kyoto, Japan), UV-VIS-NIR spectroscopy on a UV-3600i Plus spectrophotometer (Shimadzu, Kyoto, Japan), XPS on a K-Alpha X-Ray Photoelectron Spectroscopy System (Thermo Fisher Scientific, Waltham, MA, USA), and XRD on an Ultima-IV (Rigaku Corporation, Tokyo, Japan).

The surface topography of the CFRP materials and copper hydroxyphosphate additive was studied using a MIRA3 LMU scanning electron microscope (Tescan, Brno, Czech Republic). The samples were coated with a 15 nm thick Au-Pd layer using a precision coating and etching system (682 PECS, Gatan, Pleasanton, CA, USA) to reduce the surface charge. Ablation efficiency analysis used a Sensofar S neox optical profilometer with SensoVIEW 1.8.0 software (Sensofar metrology, Barcelona, Spain).

### 2.5. Ablation Efficiency Calculation

Ablation efficiency calculations were performed according to papers [35,36]. The ablation efficiency is expressed as the specific energy ΔV/ΔE_opt_ (1), defined by the following relation [47,48]:

(1) $$\frac{\Delta V}{\Delta E}=\frac{1}{2}\cdot \frac{\delta }{\phi_{0}}\cdot {ln}^{2}(\frac{\phi_{0}}{\phi_{th}}),$$

where *δ* denotes the laser energy effective penetration depth, *ϕ*_0_ represents the incident beam peak fluence, and *ϕ*_th_ is the threshold fluence required to initiate ablation.

The peak fluence—*ϕ*_0_ (2) can be defined as [49]

(2) $$\phi_{0}=\frac{2\cdot E_{p}}{\pi \cdot w_{0}^{2}},$$

where E_p_ is pulse energy (0.5–16 μJ) and w_0_ is the spot radius (~3.9 μm).

The maximum ablation rate is the optimum peak fluence *ϕ*_optimal_ (3) at removal rate as a function of laser fluence. This point corresponds to the most efficient ablation process. The ablation process loses its effectiveness if the peak fluence value is below this optimum. At higher peak fluences, the material may overheat, which reduces the quality of laser processing [50].

(3) $$\phi_{optimal}=e^{2}\cdot \phi_{th}$$

## 3. Results

### 3.1. Copper Hydroxyphosphate Characterization

**Figure 3:**
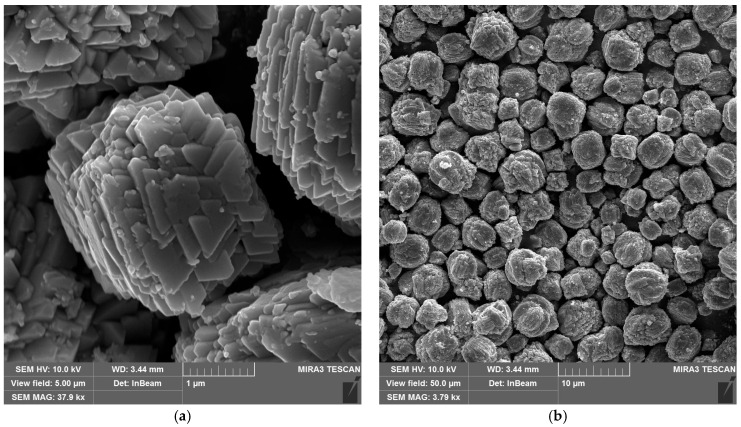
SEM images of copper hydroxyphosphate particles: (**a**) high magnification; (**b**) low magnification.

Figure 3 shows scanning electron microscope (SEM) images of the synthesized copper hydroxyphosphate particles. These particles exhibit distinct morphology in the form of cuboid microaggregates with stepped plates on their surfaces (Figure 3a). The microaggregate diameter ranges from ~1 to ~6 µm, with an average size of ~3 µm (Figure 3b). The plate size on the surface ranges from 0.4 to 0.8 µm (Figure 3a). This hierarchical structure is consistent with the anisotropic crystal growth of Cu_2_(OH)PO_4_ in the hydrothermal synthesis method described in reference [39].

The cuboid aggregate morphology with lamellar crystallites of the copper hydroxyphosphate particles should ensure the local absorption and distribution of energy during femtosecond ablation, creating local hot spots and changes in porosity and defect density. This will potentially affect the ablation threshold and the formed relief morphology [51,52].

**Figure 4:**
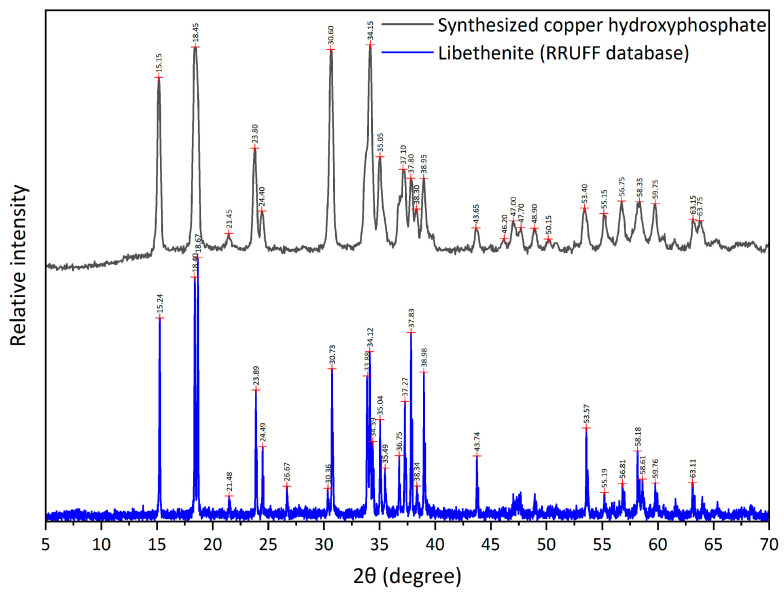
XRD of copper hydroxyphosphate.

The XRD analysis of copper hydroxyphosphate powders (Figure 4) shows the diffractogram corresponding to the diffraction pattern of libethenite (orthorhombic copper hydroxyphosphate) as reported in papers [32,39,44,53] and in databases (RRUFF Database XRD and JCPDS card No. 360404). However, for the material synthesized in this article, higher intensities are observed for the (022), (202), (311), and (122) planes.

**Figure 5:**
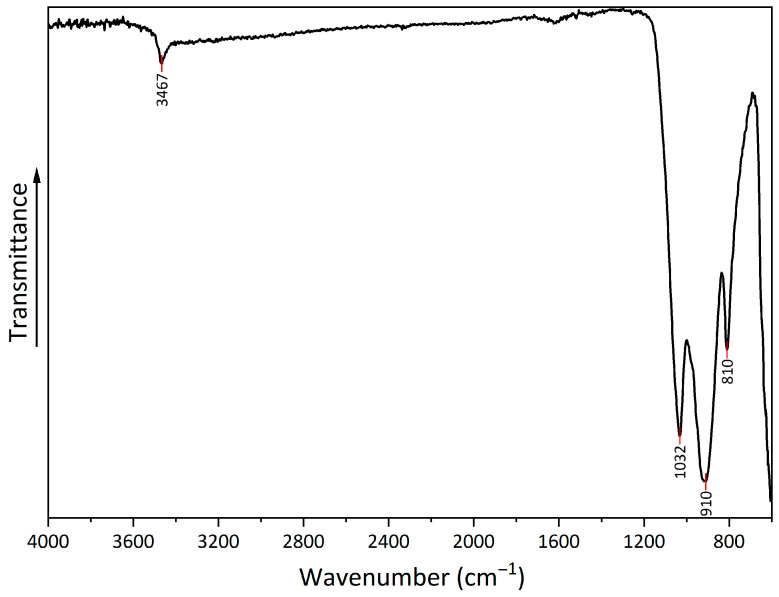
FTIR spectrum of copper hydroxyphosphate.

The FTIR spectrum (Figure 5) shows several peaks that can be attributed to copper hydroxyphosphate. At 3467 cm^−1^, there is a peak from the OH bond, which corresponds to adsorbed water. Additionally, at 1620 cm^−1^, there is a low-intensity peak, which corresponds to OH bond bending. The series of peaks at 1032, 910, and 810 cm^−1^ can be attributed to symmetric stretching vibrations of (PO_4_)^3−^ [54,55].

**Figure 6:**
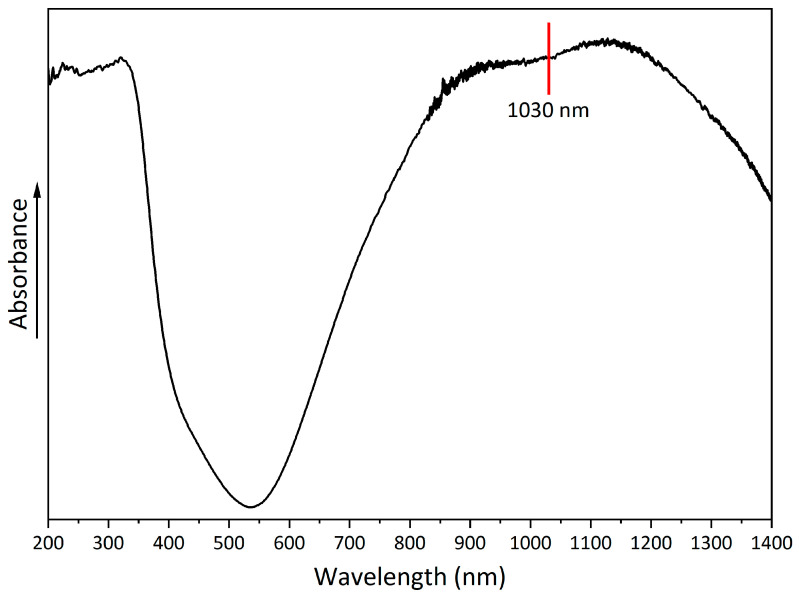
UV-Vis-NIR spectrum of copper hydroxyphosphate.

A UV-Vis-NIR spectrum was obtained to evaluate the absorption properties of copper hydroxyphosphate (Figure 6). The spectrum shows a broad absorption band with a peak at 530–540 nm. In the 850–1250 nm range, there is absorption in the near-infrared region, which indicates the possible use of copper hydroxyphosphate as an additive for absorbing laser radiation [56].

**Figure 7:**
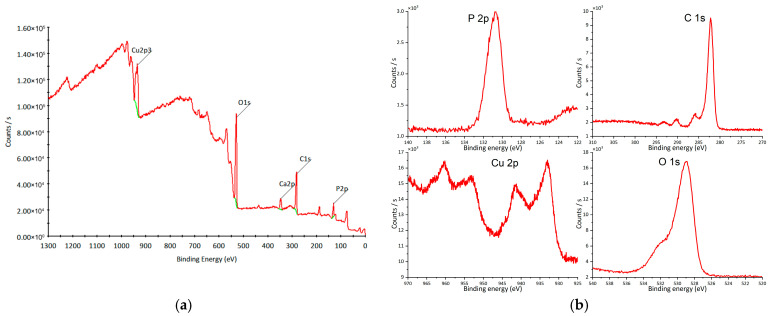
XPS of copper hydroxyphosphate: (**a**) wide spectrum; (**b**) high-resolution spectra. Red line = experimental spectrum (raw data). Green line = background curve used for correction (baseline).

XPS analysis was performed to examine the elemental composition and chemical states of O, P, and Cu (Figure 7a). The peak at 130 eV corresponds to P 2p (Figure 7b), confirming the presence of P^5+^ [57,58]. The Cu 2p region exhibits peaks at 933 eV (2p_3/2_) and 953 eV (2p_1/2_) (Figure 7b), characteristic of Cu^+^ [59], together with shake-up satellite peaks at 943 eV and 963 eV (Figure 7b), indicative of Cu^2+^ species [60,61]. The O 1s peak at 529 eV (Figure 7b) corresponds to lattice oxygen [62]. The overall spectral features are consistent with previously reported copper hydroxyapatite spectra [44,63].

### 3.2. Femtosecond Laser Ablation Efficiency

**Figure 8:**
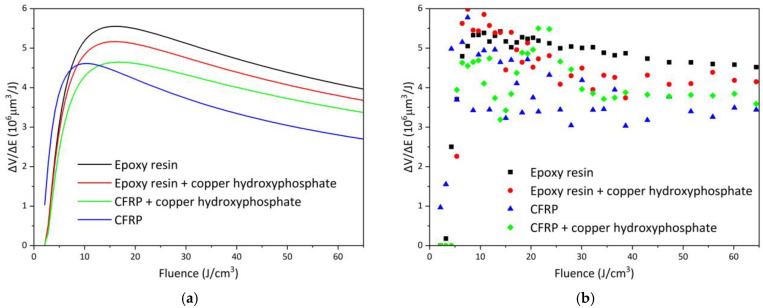
Femtosecond laser ablation efficiency of epoxy resin and CFRP with copper hydroxyphosphate: (**a**) fitted curves; (**b**) non-approximated data.

The efficiency curves of femtosecond laser ablation for epoxy and CFRP systems are presented in Figure 8, and the corresponding fitted parameters are summarized in Table 1. For neat epoxy resin, the maximum efficiency (ΔV/ΔE_opt_) of 5.55 × 106 μm^3^/J is achieved at an optimal fluence of 16.09 J/cm^3^, which is the highest among all studied samples. The addition of copper hydroxyphosphate particles to the epoxy resin reduces the efficiency to 4.65 × 106 μm^3^/J (a decrease of ~16.2%) at a similar optimal fluence of 16.76 J/cm^3^. This reduction is also reflected in the fitted energy penetration depth (δ), which decreases from 44.68 to 38.94 μm.

In contrast, neat CFRP exhibits lower ablation efficiency, reaching only 4.61 × 106 μm^3^/J at an optimal fluence of 10.31 J/cm^3^, with a penetration depth of 23.77 μm. The incorporation of copper hydroxyphosphate into CFRP, however, leads to an opposite trend: the efficiency increases by ~10.8%, reaching 5.17 × 106 μm^3^/J at an optimal fluence of 15.98 J/cm^3^, accompanied by a significant increase in δ to 41.28 μm. This indicates that copper hydroxyphosphate enhances the laser–material interaction and improves ablation process stability in heterogeneous fiber-reinforced composites.

The experimental ΔV/ΔEopt values exhibit considerable scatter, especially for CFRP samples (Figure 8b). The relative errors for the δ, φ_th_, and φ_opt_ parameters for neat epoxy are low in comparison to other samples. For neat epoxy, the fit is robust (Adj. R2 0.92, Red. Χ2 0.13, RSS 4.24), indicating good reproducibility in the homogeneous matrix. Addition of copper hydroxyphosphate to neat epoxy increases parameter uncertainties and reduces Adj. R2 to 0.73, so changes in δ, φ_th_, and φ_opt_ cannot be claimed as statistically significant under current experimental variability.

The CFRP shows a large intrinsic scatter, with Adj. R2 0.46 for neat CFRP, reflecting local variations in fiber distribution. The high RSS values for epoxy resin + copper hydroxyphosphate (19.9) and for CFRP (16.1) reflect a greater spread of experimental values around the fit model. The introduction of copper hydroxyphosphate into CFRP significantly improves model predictability (Adj. R2 increases to 0.71). These results indicate that copper hydroxyphosphate homogenizes energy absorption in the heterogeneous composite, increasing penetration depth and shifting the optimal fluence.

The non-approximated data analysis confirms that the ablation volume in CFRP strongly depends on the local fiber distribution, leading to increased variability. However, in the case of CFRP containing copper hydroxyphosphate, a slight improvement in ablation uniformity is observed (Table 1). Therefore, while the addition of copper hydroxyphosphate decreases the laser ablation efficiency of pure epoxy resin, it has a positive effect on CFRP systems, enhancing both processing uniformity and overall efficiency.

### 3.3. Morphological Changes in Materials

**Figure 9:**
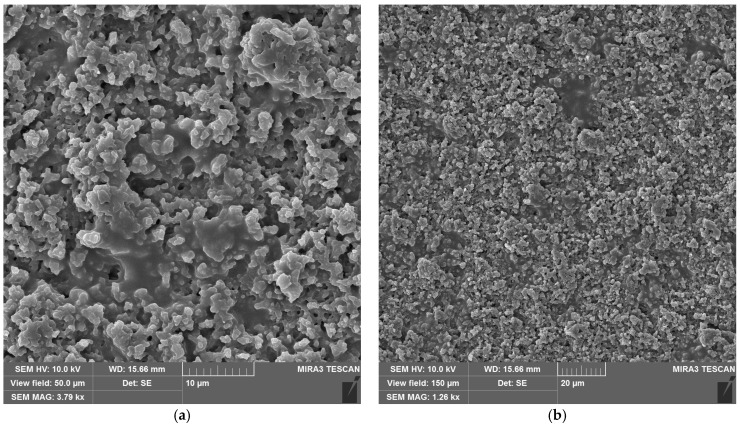

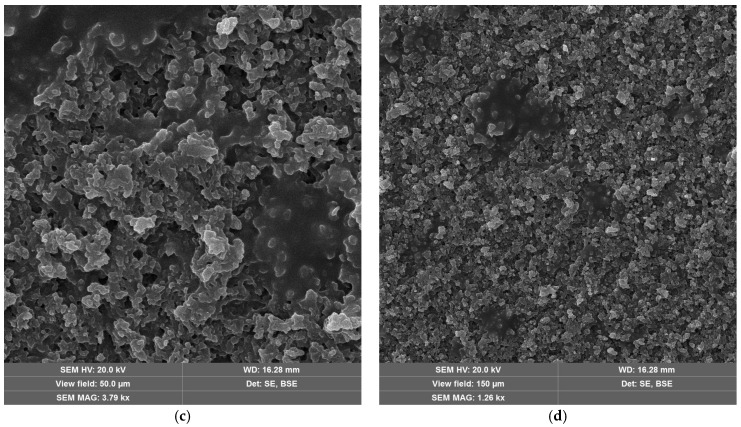
SEM images of epoxy resin after laser ablation: (**a**,**b**) neat epoxy resin; (**c**,**d**) epoxy resin + copper hydroxyphosphate.

The laser-treated-sample SEM micrographs at the optimal fluence values for maximum efficiency are shown in Figure 9. For neat epoxy resin (Figure 9a,b) and epoxy resin containing copper hydroxyphosphate (Figure 9c,d), the laser irradiation produces an irregular porous morphology on the epoxy matrix. The epoxy + copper hydroxyphosphate sample exhibits smoother regions with fewer damaged areas and reduced porosity compared to neat epoxy.

**Figure 10:**
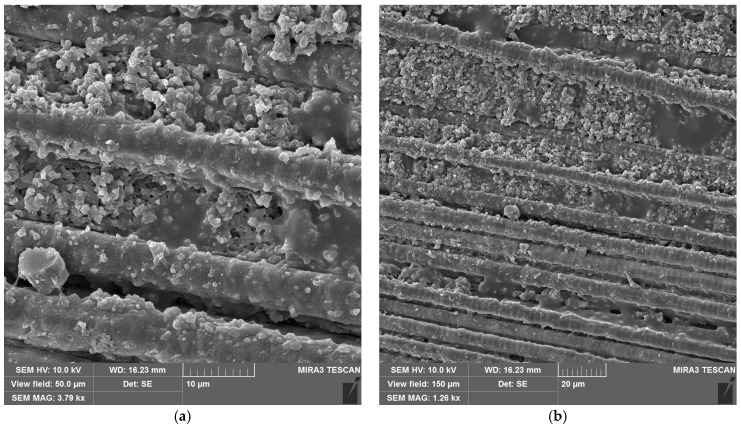

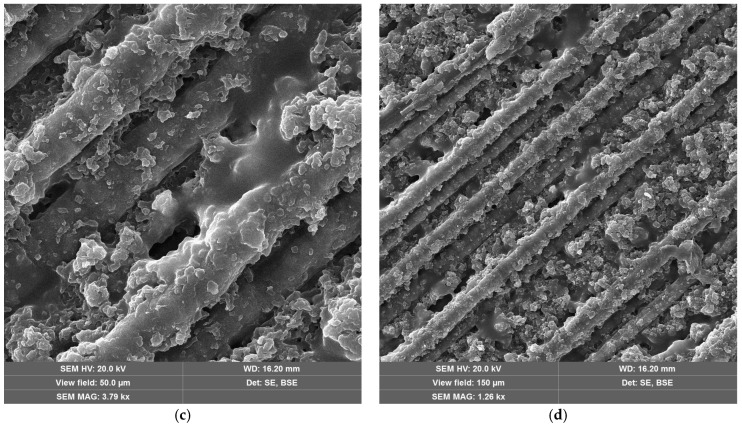
SEM images of CFRP after laser ablation: (**a**,**b**) neat CFRP; (**c**,**d**) CFRP + copper hydroxyphosphate.

SEM images of CFRP without (Figure 10a,b) and with copper hydroxyphosphate (Figure 10c,d) demonstrate that laser ablation exposes the carbon fibers. The addition of copper hydroxyphosphate leads to more uniform ablation. At low magnification, the additive-containing sample has more consistently covered fibers with epoxy resin residues, while the sample without the additive has some fibers completely exposed. Additionally, SEM images of exposed carbon fibers revealed laser-induced periodic surface structure (LIPSS) formation, consistent with previous reports [15] obtained with a 1064 nm laser. Such structures are characteristic of ultrashort-pulse laser processing and are known to depend on irradiation wavelength, incidence angle, beam polarization, and fluence [64].

## 4. Discussion

The synthesized copper hydroxyphosphate particles exhibit a hierarchical morphology, featuring cuboid microaggregates and stepped plate-like structures. The particles effectively absorb the 1030 nm laser radiation used in this study, converting it into localized heat. It is known that copper hydroxyphosphate exhibits strong visible–NIR absorption with morphology-dependent optical cross-sections and efficient photothermal conversion under continuous near-IR excitation, as shown for Cu_2_(OH)PO_4_ and related copper phosphates [65,66]. In our fs-1030 nm processing, dispersed Cu_2_(OH)PO_4_ particles act as sub-micrometer absorbers that locally convert photon energy into heat. In neat epoxy, this additional heat is dissipated into a low-conductivity matrix without efficient bond-selective photochemistry, which suppresses net material ejection and reduces ablation efficiency. In CFRP, two extra channels appear: (1) interfacial heat flows toward carbon fibers with markedly higher axial thermal conductivity than epoxy, promoting resin removal along the fiber/matrix boundary; (2) spectral/structural selectivity—carbon fibers absorb strongly in the 1 µm ranges, whereas epoxy is comparatively transparent, so Cu_2_(OH)PO_4_ increases coupling to fiber-rich regions and homogenizes matrix ejection with a limited HAZ. This picture is consistent with reports on selective resin ablation, anisotropic heat transport, and lower thresholds at fiber–matrix interfaces during ultrafast-laser processing of CFRP [15,67,68].

The introduction of copper hydroxyphosphate into the epoxy resin led to a slight decrease in ablation efficiency, reflected in the reduced penetration depth (δ) from 44.7 to 38.9 μm. This is due to incident laser energy absorption by copper hydroxyphosphate particles. Instead of contributing to volumetric material removal, part of the energy is dissipated as resin local heating and subsequent thermal decomposition. Thus, in neat epoxy resin, copper hydroxyphosphate acts as an additional energy absorber, lowering the net intensity of ablation. SEM micrographs support this interpretation: the surfaces treated with copper hydroxyphosphate show less pronounced porosity and fewer localized defects, which are distributed more evenly compared to the neat epoxy. This correlation between morphology and reduced efficiency highlights a trade-off between surface quality and volumetric removal rate.

For neat CFRP, ablation efficiency remained relatively low, with a penetration depth of ~23.8 μm. This is consistent with the composite heterogeneous nature: carbon fibers and the epoxy matrix differ in their optical absorption and thermal responses, which leads to highly variable ablation behavior [69,70,71]. Such structural heterogeneity is one of the key limitations for stable femtosecond laser processing of CFRPs.

Adding copper hydroxyphosphate to CFRP increased penetration depth to 41.3 μm, while the scatter in efficiency values was reduced. This suggests that copper hydroxyphosphate modifies the interaction between the epoxy matrix and the carbon fibers, enabling more uniform absorption of laser energy. As a result, the ablation process becomes more stable and less dependent on local fiber distribution. SEM images confirm this effect: in CFRP + copper hydroxyphosphate samples, carbon fibers are less frequently fully exposed, and resin residues remain more consistently covering the fibers. This indicates that copper hydroxyphosphate facilitates controlled and homogeneous matrix removal, which directly translates into higher-quality cuts.

Overall, copper hydroxyphosphate is an effective absorber of laser energy; its influence depends on the system. In pure epoxy, it reduces ablation efficiency but improves surface quality. In CFRP, it plays a more critical role: it homogenizes the energy distribution, mitigates the heterogeneity of fiber–matrix interactions, and enhances penetration depth and uniformity of ablation. These results demonstrate that incorporating copper hydroxyphosphate can improve the precision and quality of femtosecond laser processing of CFRPs, offering efficient and defect-free laser cutting of advanced composites.

## 5. Conclusions

Copper hydroxyphosphate particles were successfully synthesized in the form of cuboid aggregates with stepped plates. Structural analysis confirmed the phase as copper hydroxyphosphate–libethenite, and optical characterization demonstrated its ability to absorb laser radiation at 1030 nm. In epoxy resin, copper hydroxyphosphate reduced the ablated volume (−16% efficiency) and penetration depth due to enhanced surface absorption and localized heating. Laser-treated surfaces exhibited smoother morphology, reduced porosity, and more uniform matrix degradation. For CFRP, the additive had a positive effect: ablation efficiency and penetration depth increased, while data variability decreased. SEM observations confirmed more homogeneous matrix removal and better preservation of resin residues on carbon fibers, which resulted in improved process stability and cut quality. Overall, copper hydroxyphosphate serves as an effective laser-absorbing additive, enhancing the uniformity and accuracy of CFRP laser cutting and contributing to the development of advanced composite machining technologies.

## Figures and Tables

**Table 1:** Fitted data summary.

Parameter	Neat Epoxy Resin	Epoxy Resin + Copper Hydroxyphosphate	Neat CFRP	CFRP + Copper Hydroxyphosphate
Adjusted R^2^	0.92	0.73	0.46	0.71
Reduced χ^2^	0.13	0.62	0.50	0.52
RSS	4.24	19.9	16.1	16.7
δ	44.68 ± 1.6	41.28 ± 3.4	23.77 ± 2.0	38.94 ± 3.2
*ϕ*_th_	2.18 ± 0.07	2.16 ± 0.17	1.39 ± 0.12	2.27 ± 0.18
*ϕ*_optimal_	16.09 ± 0.5	15.98 ± 1.3	10.31 ± 0.9	16.76 ± 1.3
ΔV/ΔE_opt_	5.55	5.17	4.61	4.65

## Data Availability

The original contributions presented in this study are included in the article. Further inquiries can be directed to the corresponding author.

## Abbreviations

The following abbreviations are used in this manuscript:

CFRP	Carbon-fiber-reinforced plastic
HAZ	Heat-affected zone
fs	Femtosecond
FTIR	Fourier-transform infrared spectroscopy
XPS	X-ray photoelectron spectroscopy
XRD	X-ray diffraction
UV-VIS-NIR	Ultraviolet/visible/near-infrared spectroscopy

## Appendix B

The EDS analysis (Table A1) shows changes in the surface composition of epoxy- and CFRP-based samples before and after femtosecond laser ablation. For neat epoxy, ablation increases carbon (from 57.1% to 59.1%) and decreases oxygen (from 29.6% to 26.1%) and nitrogen (from 13.3% to 11.3%). This suggests polymer matrix decomposition and oxygen loss due to localized heating [72,73]. In epoxy resin containing copper hydroxyphosphate, the surface after ablation shows an increase in carbon (from 63.3% to 65.9%) and a decrease in oxygen (from 26.9% to 24.8%), similar to a neat epoxy sample. Copper content slightly decreases from 1.9% to 1.6%, indicating redistribution or possible removal of copper hydroxyphosphate particles during ablation.

For neat CFRP, laser treatment reduces the nitrogen concentration significantly (from 13.6% to 9.5%), while oxygen slightly increases (from 25.8% to 27.7%). The carbon fraction remains almost unchanged, consistent with exposure of carbon fibers and the surrounding epoxy matrix [74]. In CFRP with a copper hydroxyphosphate additive, carbon content remains stable (from 63.8% to 64.0%), whereas oxygen decreases (from 26.6% to 25.5%). At the same time, copper increases from 1.3% to 1.8% after ablation, which may be related to better retention or exposure of copper hydroxyphosphate particles on the surface under the influence of femtosecond laser radiation. The data show that femtosecond laser processing increases carbon and reduces oxygen in epoxy-based systems, while in CFRP, the process is influenced by fiber exposure and copper hydroxyphosphate redistribution.

**Table A1:** EDS analysis of laser-ablated areas.

Sample	Laser Processing	Element				
		C	N	O	P	Cu
Neat epoxy resin	before	57.1	13.3	29.6		
	after	59.1	11.3	26.1		
Epoxy resin + copper hydroxyphosphate	before	63.3	7.5	26.9	0.5	1.9
	after	65.9	7.2	24.8	0.5	1.6
Neat CFRP	before	60.6	13.6	25.8		
	after	61.4	9.5	27.7		
CFRP + copper hydroxyphosphate	before	63.8	7.8	26.6	0.4	1.3
	after	64.0	8.2	25.5	0.5	1.8

## References

[1] Karataş M.A., Gökkaya H. (2018). A Review on Machinability of Carbon Fiber Reinforced Polymer (CFRP) and Glass Fiber Reinforced Polymer (GFRP) Composite Materials. Def. Technol..

[2] Sharma H., Kumar A., Rana S., Guadagno L. (2022). An Overview on Carbon Fiber-Reinforced Epoxy Composites: Effect of Graphene Oxide Incorporation on Composites Performance. Polymers.

[3] Hegde S., Shenoy B.S., Chethan K.N. (2019). Review on Carbon Fiber Reinforced Polymer (CFRP) and Their Mechanical Performance. Mater. Today Proc..

[4] Song G.L., Zhang C., Chen X., Zheng D. (2021). Galvanic Activity of Carbon Fiber Reinforced Polymers and Electrochemical Behavior of Carbon Fiber. Corros. Commun..

[5] Vijayan D.S., Sivasuriyan A., Devarajan P., Stefańska A., Wodzyński Ł., Koda E. (2023). Carbon Fibre-Reinforced Polymer (CFRP) Composites in Civil Engineering Application—A Comprehensive Review. Buildings.

[6] Pawlak A.M., Górny T., Dopierała Ł., Paczos P. (2022). The Use of CFRP for Structural Reinforcement—Literature Review. Metals.

[7] Atescan-Yuksek Y., Mills A., Ayre D., Koziol K., Salonitis K. (2024). Comparative Life Cycle Assessment of Aluminium and CFRP Composites: The Case of Aerospace Manufacturing. Int. J. Adv. Manuf. Technol..

[8] Jaśkiewicz R. (2019). Comparison of Composite Laminates Machining Methods and Its Influence on Process Temperature and Edge Quality. Trans. Aerosp. Res..

[9] Kartal F. (2025). Abrasive Water Jet Machining of Carbon Fiber-Reinforced PLA Composites: Optimization of Machinability and Surface Integrity for High-Precision Applications. Polymers.

[10] Jiao J., Cheng X., Wang J., Sheng L., Zhang Y., Xu J., Jing C., Sun S., Xia H., Ru H. (2022). A Review of Research Progress on Machining Carbon Fiber-Reinforced Composites with Lasers. Micromachines.

[11] Chen M., Guo B., Jiang L., Liu Z., Qian Q. (2023). Analysis and Optimization of the Heat Affected Zone of CFRP by Femtosecond Laser Processing. Opt. Laser Technol..

[12] Zhao C., Ma Z., Sun J., Zhu L. (2023). Femtosecond Laser Drill High Modulus CFRP Multidirectional Laminates with a Segmented Arc-Based Concentric Scanning Method. Compos. Struct..

[13] Jiang H., Ma C., Li M., Cao Z. (2021). Femtosecond Laser Drilling of Cylindrical Holes for Carbon Fiber-Reinforced Polymer (CFRP) Composites. Molecules.

[14] Chen J., Li Y., Huang M., Dong L. (2023). Comparison of the Effects of Femtosecond and Nanosecond Laser Tailoring on the Bonding Performance of the Heterojunction between PEEK/CFRP and Al–Li Alloy. Int. J. Adhes. Adhes..

[15] Gebauer J., Burkhardt M., Franke V., Lasagni A.F. (2020). On the Ablation Behavior of Carbon Fiber-Reinforced Plastics during Laser Surface Treatment Using Pulsed Lasers. Materials.

[16] Wang Z., Ma Y., Yuan B., Wu C., Li C., Sun S. (2023). Development of Laser Processing Carbon-Fiber-Reinforced Plastic. Sensors.

[17] Fujita M., Ohkawa H., Somekawa T., Otsuka M., Maeda Y., Matsutani T., Miyanaga N. (2016). Wavelength and Pulsewidth Dependences of Laser Processing of CFRP. Phys. Procedia.

[18] Sharma S.P., Vilar R. (2022). Femtosecond Laser Micromachining of Carbon Fiber-Reinforced Epoxy Matrix Composites. J. Manuf. Process..

[19] Zhang Z., Zhou J., Ren Y., Li W., Li S., Chai N., Zeng Z., Chen X., Yue Y., Zhou L. (2022). Passive Deicing CFRP Surfaces Enabled by Super-Hydrophobic Multi-Scale Micro-Nano Structures Fabricated via Femtosecond Laser Direct Writing. Nanomaterials.

[20] Zuo P., Liu T., Li F., Wang G., Zhang K., Li X., Han W., Tian H., Hu L., Huang H. (2024). Controllable Fabrication of Hydrophilic Surface Micro/Nanostructures of CFRP by Femtosecond Laser. ACS Omega.

[21] Zhang J., Bi R., Jiang S., Wen Z., Luo C., Yao J., Liu G., Chen C., Wang M. (2022). Laser Ablation Mechanism and Performance of Carbon Fiber-Reinforced Poly Aryl Ether Ketone (PAEK) Composites. Polymers.

[22] Xu L.Y., Lu J.R., Li K.M., Hu J. (2021). Experimental Study of CFRP Laser Surface Modification and Bonding Characteristics of CFRP/Al6061 Heterogeneous Joints. Compos. Struct..

[23] Kononenko T.V., Freitag C., Komlenok M.S., Onuseit V., Weber R., Graf T., Konov V.I. (2014). Oxygen-Assisted Multipass Cutting of Carbon Fiber Reinforced Plastics with Ultra-Short Laser Pulses. J. Appl. Phys..

[24] Elkington H., Diboine J., Chingwena K., Mason B., Marimuthu S. (2023). Water Jet Guided Nanosecond Laser Cutting of CFRP. Opt. Laser Technol..

[25] Cui K., Han X., Zhou P., Hao M., Wang X., Bian L., Nie J., Yang G., Liang J., Liu X. (2024). A Novel Highly Dispersed Calcium Silicate Hydrate Nanosheets for Efficient High-Concentration Cu^2+^ Adsorption. J. Hazard. Mater..

[26] Stock J., Zaeh M.F., Conrad M. (2012). Remote Laser Cutting of CFRP: Improvements in the Cut Surface. Phys. Procedia.

[27] Pérez-Barrado E., Darton R.J. (2018). Synthesis and Applications of Near-Infrared Absorbing Additive Copper Hydroxyphosphate. MRS Commun..

[28] Cheng J., Zhou J., Zhang C., Cao Z., Wu D., Liu C., Zou H. (2019). Enhanced Laser Marking of Polypropylene Induced by “Core-Shell” ATO@PI Laser-Sensitive Composite. Polym. Degrad. Stab..

[29] Cheng J., Li H., Zhou J., Cao Z., Wu D., Liu C. (2018). Influences of Diantimony Trioxide on Laser-Marking Properties of Thermoplastic Polyurethane. Polym. Degrad. Stab..

[30] Sobotova L., Badida M. (2017). Laser Marking as Environment Technology. Open Eng..

[31] Shridharan T.S., Lee J.H., Tan R., Sivanantham A., Han H.S., Jung H.S., Cho I.S. (2024). Unique Photothermal Material: Copper Phosphate (Cu_3_P_2_O_8_) with Broadband Visible-to-near-Infrared Absorption Properties for Efficient Solar Steam Generation. Desalination.

[32] Cho I., Kim D.W., Lee S., Kwak C.H., Bae S., Noh J.H., Yoon S.H., Jung H.S., Kim D., Hong K.S. (2008). Synthesis of Cu_2_PO_4_OH Hierarchical Superstructures with Photocatalytic Activity in Visible Light. Adv. Funct. Mater..

[33] Han D., Meng Z., Wu D., Zhang C., Zhu H. (2011). Thermal Properties of Carbon Black Aqueous Nanofluids for Solar Absorption. Nanoscale Res. Lett..

[34] Guo X., Cheng S., Cai W., Zhang Y., Zhang X.-A. (2021). A Review of Carbon-Based Thermal Interface Materials: Mechanism, Thermal Measurements and Thermal Properties. Mater. Des..

[35] Solati E., Aghazadeh Z., Dorranian D. (2019). Effects of Liquid Ablation Environment on the Characteristics of TiO_2_ Nanoparticles. J. Clust. Sci..

[36] Torres P., Rurali R. (2019). Thermal Conductivity of Rutile and Anatase TiO_2_ from First-Principles. J. Phys. Chem. C.

[37] Zhan Z., Sun W., Zhang Z., Xiong X., Xu Y., Zeng Y., Yin J. (2019). Properties of −O–CU–O– Bridged Copper Phosphate-Based Thermal Insulation Materials. ACS Omega.

[38] Fu W., Wang R., Wu L., Wang H., Wang X., Wang A., Zhang Z., Qiu S. (2013). Synthesis of Cu_2_(OH)PO_4_ Crystals with Various Morphologies and Their Catalytic Activity in Hydroxylation of Phenol. Chem. Lett..

[39] Xu J., Xue D. (2006). Fabrication of Copper Hydroxyphosphate with Complex Architectures. J. Phys. Chem. B.

[40] Zhao Y., Teng F., Xu J., Liu Z., Yang Y., Zhang Q., Yao W. (2015). Facile Synthesis of Cu_2_PO_4_OH Hierarchical Nanostructures and Their Improved Catalytic Activity by a Hydroxyl Group. RSC Adv..

[41] Xu J., Zhang J., Liu X. (2012). Hydrothermal Synthesis of Copper Hydroxyphosphate Hierarchical Architectures. Chem. Eng. Technol..

[42] Othmani M., Bachoua H., Ghandour Y., Aissa A., Debbabi M. (2017). Synthesis, Characterization and Catalytic Properties of Copper-Substituted Hydroxyapatite Nanocrystals. Mater. Res. Bull..

[43] Bai J., Hao M., Han X., Zhou P., Yao H., Bian L., Yang G., Liang J., Laine R.M., Wang F. (2024). Halloysite-Derived Hierarchical Cobalt Silicate Hydroxide Hollow Nanorods Assembled by Nanosheets for Highly Efficient Electrocatalytic Oxygen Evolution Reaction. J. Mater. Sci. Technol..

[44] Xu Y., Jiao X., Chen D. (2011). Hydrothermal Synthesis and Characterization of Copper Hydroxyphosphate Hierarchical Superstructures. J. Dispers. Sci. Technol..

[45] Hu C., Li P., Zhang W., Che Y., Sun Y., Chi F., Ran S., Liu X., Lv Y. (2017). Effect of Cupric Salts (Cu(NO_3_)_2_, CuSO_4_, Cu(CH_3_COO)_2_) on Cu_2_(OH)PO_4_ Morphology for Photocatalytic Degradation of 2,4-Dichlorophenol under Near-Infrared Light Irradiation. Mater. Res..

[46] Šlevas P., Minkevičius J., Ulčinas O., Orlov S., Vanagas E., Bilousova A., Baklan D., Myronyuk O. (2025). An Investigation of Carbon-Fiber-Reinforced Plastic Ablation by Femtosecond Laser Pulses for Further Material Cutting. Coatings.

[47] Förster D.J., Jäggi B., Michalowski A., Neuenschwander B. (2021). Review on Experimental and Theoretical Investigations of Ultra-Short Pulsed Laser Ablation of Metals with Burst Pulses. Materials.

[48] Neuenschwander B., Kramer T., Lauer B., Jaeggi B. (2015). Burst Mode with Ps- and Fs-Pulses: Influence on the Removal Rate, Surface Quality, and Heat Accumulation. Proc. SPIE.

[49] Mannion P., Magee J., Coyne E., O’Connor G.M. (2003). Ablation Thresholds in Ultrafast Laser Micromachining of Common Metals in Air. Proc. SPIE.

[50] Jaeggi B., Neuenschwander B., Zimmermann M., Penning L., deLoor R., Weingarten K., Oehler A. (2014). High-Throughput and High-Precision Laser Micromachining with Ps-Pulses in Synchronized Mode with a Fast Polygon Line Scanner. Proc. SPIE.

[51] Wu D., Peng J., Cai Z., Weng J., Luo Z., Chen N., Xu H. (2015). Gold Nanoparticles as a Saturable Absorber for Visible 635 Nm Q-Switched Pulse Generation. Opt. Express.

[52] Alrebdi T.A., Sadiq S., Tian S.C., Asghar M., Saghir I., Asghar H. (2025). Applications of Prepared MnMoO4 Nanoparticles as Saturable Absorbers for Q-Switched Erbium-Doped Fiber Lasers: Experimental and Theoretical Analysis. Photonics.

[53] Zhan Y., Li H., Chen Y. (2010). Copper Hydroxyphosphate as Catalyst for the Wet Hydrogen Peroxide Oxidation of Azo Dyes. J. Hazard. Mater..

[54] Yuan A.Q., Liao S., Tong Z.F., Wu J., Huang Z.Y. (2006). Synthesis of Nanoparticle Zinc Phosphate Dihydrate by Solid State Reaction at Room Temperature and Its Thermochemical Study. Mater. Lett..

[55] Klähn M., Mathias G., Kötting C., Nonella M., Schlitter J., Gerwert K., Tavan P. (2004). IR Spectra of Phosphate Ions in Aqueous Solution: Predictions of a DFT/MM Approach Compared with Observations. J. Phys. Chem. A.

[56] Helm J., Schulz A., Olowinsky A., Dohrn A., Poprawe R. (2020). Laser Welding of Laser-Structured Copper Connectors for Battery Applications and Power Electronics. Weld. World.

[57] Wu X., Gong K., Zhao G., Lou W., Wang X., Liu W. (2018). Mechanical Synthesis of Chemically Bonded Phosphorus–Graphene Hybrid as High-Temperature Lubricating Oil Additive. RSC Adv..

[58] Wang Z., Shang Y., Chen H., Cao S., Zhu Q., Liu S., Wei S., Lu X. (2023). Toward Highly Active Electrochemical CO2 Reduction to C2H4 by Copper Hydroxyphosphate. J. Solid State Electrochem..

[59] Biesinger M.C. (2017). Advanced Analysis of Copper X-ray Photoelectron Spectra. Surf. Interface Anal..

[60] Tang Z., Pan Y., Zhao Q., Cao Y., Su C., Gao P., Liu Z., Chen Y., Li G., Wang Q. (2024). Room-Temperature Synthesis of Nonstoichiometric Copper Sulfide (Cu2−xS) for Sodium Ion Storage. Inorg. Chem. Front..

[61] De Sousa P.V.F., De Oliveira A.F., Da Silva A.A., Lopes R.P. (2019). Environmental Remediation Processes by Zero Valence Copper: Reaction Mechanisms. Environ. Sci. Pollut. Res..

[62] Sydorchuk V., Poddubnaya O.I., Tsyba M.M., Zakutevskyy O., Khyzhun O., Khalameida S., Puziy A.M. (2020). Photocatalytic Degradation of Dyes Using Phosphorus-Containing Activated Carbons. Appl. Surf. Sci..

[63] Pan M.Y., Lu S.T., Li Y.Y., Li C., Cao K.Z., Fan Y. (2023). Copper Hydroxyphosphate Cu_2_(OH)PO_4_ as Conversion-Type Anode Material for Lithium-Ion Batteries. Ionics.

[64] Oliveira V., Sharma S.P., De Moura M.F.S.F., Moreira R.D.F., Vilar R. (2017). Surface Treatment of CFRP Composites Using Femtosecond Laser Radiation. Opt. Lasers Eng..

[65] Kwak C.H., Cho I.S., Lee S., An J.S., Hong K.S. (2010). Hydrothermal Synthesis, Characterization and Photocatalytic Properties of Cu2PO4OH with Hierarchical Morphologies. J. Nanosci. Nanotechnol..

[66] Hu X., Zheng X.-J., Li Y., Zhang J., Ma D.-K. (2016). Cu_2_PO_4_OH: Controlled Synthesis of Various Architectures and Morphology-Dependent 808 Nm Laser-Driven Photothermal Performance. J. Alloys Compd..

[67] Lebeda F., Demleitner M., Pongratz A., Ruckdäschel H., Retsch M. (2024). Shaping Thermal Transport and Temperature Distribution via Anisotropic Carbon Fiber Reinforced Composites. ACS Omega.

[68] Wang B., Yan Y., Qin B., Ye Z., Cao J., Qi J. (2023). Design of Efficient Thermal Conductive Epoxy Resin Composites via Highspeed Transport Pathways of Heterogeneous Compatible Carbon Framework. J. Chem. Eng..

[69] ALYousef J., Yudhanto A., Tao R., Lubineau G. (2022). Laser Ablation of CFRP Surfaces for Improving the Strength of Bonded Scarf Composite Joints. Compos. Struct..

[70] Hou Y., Bai J., Wang F., Qian L. (2023). Performance and Mechanisms of Ultraviolet Laser Ablation of Plain-Woven CFRP Composites. Compos. Struct..

[71] Li S., Wang L., Yang W., Wang W., Wang D., Li Z., Teng Z. (2023). Layer Ablation and Surface Textured of Carbon Fiber Reinforced Plastics by Infrared Pulsed Laser. Polym. Compos..

[72] Zhumanazarova G.M., Sarsenbekova A.Z., Abulyaissova L.K., Figurinene I.V., Zhaslan R.K., Makhmutova A.S., Sotchenko R.K., Aikynbayeva G.M., Hranicek J. (2025). Study of Mathematical Models Describing the Thermal Decomposition of Polymers Using Numerical Methods. Polymers.

[73] Ray S., Cooney R.P. (2012). Thermal Degradation of Polymer and Polymer Composites.

[74] Yatim N.M., Shamsudin Z., Shaaban A., Sani N.A., Jumaidin R., Shariff E.A. (2020). Thermal Analysis of Carbon Fibre Reinforced Polymer Decomposition. Mater. Res. Express.

